# Positive influence of aqua exercise and burdock extract intake on fitness factors and vascular regulation substances in elderly

**DOI:** 10.3164/jcbn.18-60

**Published:** 2018-10-13

**Authors:** Min-Seong Ha, Ji-Hyeon Kim, Soo-Min Ha, You-Sin Kim, Do-Yeon Kim

**Affiliations:** 1 Laboratory of Exercise Biochemistry and Neuroendocrinology, Faculty of Health and Sports Sciences, University of Tsukuba, 1-1-1 Tennoudai, Tsukuba, Ibaraki 305-8574, Japan; 2 Laboratory of Exercise Physiology, Department of Physical Education, Pusan National University, 2 Busandaehak-ro 63beon-gil, Geumjeong-gu, Busan 46241, Republic of Korea; 3 Department of Leisure Sports, Jungwon University, 85 Munmu-ro, Goesan-eup, Goesan-gun, Chungbuk 28024, Republic of Korea

**Keywords:** aqua exercise, burdock extract intake, senior fitness, PGI_2__,_ TXA_2_

## Abstract

Health issues in elderly individuals are often complex and tend to lead to chronic diseases; such issues can be due to a decline in fitness resulting from lack of physical activity. Aqua exercise and burdock are positive effects on cardiovascular disease and vascular health. This study investigated the changes due to aqua exercise and burdock extract intake in senior fitness, prostaglandin I_2_ (PGI_2_), and thromboxane A_2_ (TXA_2_) in elderly women. Forty elderly women (65–80 years) volunteered for this study. After baseline measurements, participants were randomized into control (*n* = 8), aqua exercise (*n* = 11), aqua exercise and burdock extract intake combination (*n* = 11), and burdock extract intake groups (*n* = 10). The variables of senior fitness tests, PGI_2_ and TXA_2_ were measured in all participants before and after the 12-week study. Blood collections were carried out at the beginning- and the end of aqua exercise training. Muscular strength, endurance, flexibility, and cardiorespiratory endurance of aqua exercise and burdock extract intake group at post-test significantly increased compared to pre-test (*p*<0.05). There were no significant differences in PGI_2_ and TXA_2_ between pre- and post-training programs. In conclusion, our findings indicated that the aqua exercise and burdock extract intake improves senior fitness factors in elderly Korean women. Also, the program participation led to a balance between PGI_2_ and TXA_2_. Additionally, burdock extract intake may be useful in vascular health by playing a secondary role in disease prevention and health promotion.

## Introduction

Society is currently witnessing a sharp growth in the elderly population as the average lifespan increases due to advances in medical and healthcare technologies and improvements in living standards.^(1)^ South Korea is fast approaching an aging society phase; 9.5% the population was aged 65 and older in 2006, and that is expected to be 14.3% in 2018 and 20.8% in 2026, entering the aging and ultra-aging society phases, respectively, based, on the United Nations’ standard.^(2)^

Health issues in elderly individuals are often complex and tend to lead to chronic diseases,^(3)^ such issues can be due to a decline in fitness resulting from lack of physical activity.^(4)^ According to the Sports for All General Survey conducted by the Korea Ministry of Culture, Sports, and Tourism,^(5)^ the percentage of elderly individuals in their 60s and 70s who engage in regular physical exercise is merely half that of those in their 50s.

Synthesized by prostaglandin and generated in the vessel wall, prostaglandin I_2_ (PGI_2_) is a type of eicosanoid that delivers hormone-like signals, although it is not a hormone.^(6–8)^ PGI_2_ is a bioactive substance synthesized in animal tissues. It is closely related to diseases caused by tissue damage, such as the damaged inner wall of an artery, due to its clearly bioactive features such as enlarging blood vessels, lowering blood pressure, and inhibiting platelet aggregation,^(9–12)^ and maintaining the physiological state of the circulatory system through antagonizing thromboxaneA_2_ (TXA_2_).^(13,14)^ TXA_2_ is a type of bioactive substance synthesized with animal tissues in eicosatetraenoic acid (ETA) and has been found to induce platelet aggregation, bronchoconstriction, and vasoconstriction.^(13,15)^

In addition, although it maintains homeostasis within blood vessels due to mutually antagonistic action with PGI_2_ generated in the vascular endothelial cells in the normal state, TXA_2_ facilitates platelet aggregation and clotting in the state of imbalance with PGI_2_ caused by a chemical reaction to a physical stimulus in the body.^(10,16)^ Regarding research on PGI_2_ and TXA_2_, their clinical significance and relationship with endocrine diseases have been studied extensively; however, little research has been conducted on the effects of aging and regular exercise on the bioactive substances in the context of vascular health.

The American College of Sports Medicine (ACSM)^(17)^ recommends aqua exercise as an effective exercise for maintaining and improving muscular strength and physique. Studies show evidence of the positive effects of aqua exercise on cardiovascular and metabolic diseases as well as other positive effects among people with obesity or musculoskeletal disease and elderly individuals.^(18,19)^

Burdock is an alkali food with sugar as the main ingredient, 76% water content, high fiber content, and low vitamin content.^(20,21)^ Burdock also cleans the blood; lowers body temperature; heals bronchial disease, removes furuncles and toxins; heals weakness of limbs, stroke, beriberi, and visceral pain;^(21–23)^ and helps excretion of cholesterol and fat, having a positive effect on cardiovascular disease and vascular health.^(21,24)^

Therefore, the objective of this study is to determine the key factors that influence functional fitness, PGI_2_, and TXA_2_ in elderly women by implementing a 12-week program of aqua exercise and/or burdock extract intake.

## Material and Methods

### Participants

Forty elderly women (65–80 years) volunteered for this study. Participants were sedentary at baseline, defined as having no regular structured exercise or physical activity. The health of the participants was determined using a health questionnaire, physical examination, and laboratory tests. All participants provided written informed consent approved by the Institutional Human Research Committee. The purpose, goal, and experimental procedures were thoroughly outlined verbally to each participant. Prior to their participation, they were informed about the possible risks and discomforts involved in the study.

### Study design

After prescreening and recruitment, eligible participants attended a study meeting in which all procedures were explained. Participants were tested in the morning at the same time each day to avoid diurnal variations of temperature. Participants were asked to refrain from exercising 24 h before testing and from caffeine or alcohol ingestion the day before or the morning of each test, to otherwise follow their normal diet, and to eat a light meal 2 h before coming to the laboratory. Upon arrival, a 12 h fasting blood sample, data from a standardized health questionnaire, and anthropometry were obtained. After their baseline measurements, participants were randomized into control group (CG; *n* = 8), aqua exercise group (AEG; *n* = 11), aqua exercise and burdock extract intake combination group (AEBG; *n* = 11), and burdock extract intake group (BG; *n* = 10).

The AEBG and BG participants were instructed to not take health supplements other than the burdock extracts that were part of the experimental regimen during the 12 week experimental period.

### Anthropometric assessments

Height was measured to the nearest 0.1 cm with the participants barefoot. Weight was measured to the nearest 0.1 kg with light clothes. From these measurements, body mass index was calculated as weight in kilograms divided by the square of height in meters (kg/m^2^).

### Senior fitness tests

Senior Fitness Tests, including aerobic endurance (walking ability test, 6 min walk), upper body strength (grip strength test), lower body strength (30 s chair stand), flexibility (sit-and-reach test) and agility (8-fit-up-and-go) was measured at pre and post the aqua exercise program. All the tests have high reliability and validity.

### Blood sampling

All blood obtained after the experiment was collected in an anticoagulant tube, and 50 µl of detection reagent A was added to a plate coated with PGI_2_ reagent using an enzyme immunoassay kit (Amersham, Inc., Cleveland, OH), and reacted at 37°C for 1 h. After completion of the reaction, washing was performed four times with 350 µl of 1× wash buffer. After removing the buffer, 100 µl of all detection reagent B was added and reacted at 37°C for 30 min. After the reaction was completed, 350 µl of 1× buffer was washed four times. After removing the buffer, 90 µl of the substrate solution was added to all wells, and the reaction was carried out at room temperature for 15 to 25 min. At the end of the reaction, 50 µl of stop solution was added to all wells, and 450 nm were measured on mainfold-24 (Amersham, Inc.).

For the TXA_2_ measurement, 0.9 ml of blood was taken and immediately placed in a polystyrene tube; 0.1 ml of 3.8% trisodium citrate was then added followed by 1 ml of physiological saline. Collagen was added at a concentration of 2 µl/ml and then stimulated with TXA_2_ for 15 min. The mixture was centrifuged at 2,000 rpm for 5 min. Then, the supernatant was taken and quantified with thromboxane B2 [3H] radioimmunoassay kit (Amersham, Inc., Cleveland, OH) as an unstable TXA_2_ conversion.

### Aqua exercise program

The aqua exercise program was designed by modifying the water exercise for seniors guideline,^(25)^ and the average water temperature in the swimming pool was maintained at 26–28°C. Considering that the study participants were elderly women aged 65–80 years old, the program was implemented three times a week for 12 weeks after a 1–2-week adjustment period. Each session lasted for 50 min, consisting of a 5 min warm-up, a 40 min main exercise period, and a 5 min wrap-up. Exercise intensity was measured using the rating of perceived exertion (RPE): RPE 9–10 for Weeks 1–4, RPE 11–12 for Weeks 4–8, and RPE 13–14 for Weeks 9–12.^(26)^ Changes in heart rate were also measured with the Polar, a wristwatch-type heart rate measurement device, with a target heart rate of 30–60% heart rate reserve during exercise.

### Ingredient and intake

Burdock extracts were obtained by washing the burdock roots harvested in the Sancheong region of Gyeongnam province with water, cutting them into pieces, drying them in the sun, and boiling them for 3 h. The extracts were placed into 100 ml plastic packs, sealed, and given to the study participants. They were instructed to consume 100 ml of extract after breakfast, lunch, and supper daily for a total intake of 300 ml per day. The main ingredients of burdock extract can be found in Table 1.

### Statistical analysis

Before conducting the research, we checked the sample size needed using a priori power analysis with the statistical software G-Power.^(27)^ An optimal total sample size of *n* = 38, with a medium-large effect size of [β] = 0.2 and a power of 0.5 and alpha = 0.05 was calculated. All data were presented as a mean ± SD, and all statistical analyses were completed using the Statistical Package for Social Sciences (SPSS) ver. 23.0 for Windows (SPSS Inc., Chicago, IL). All statistical tests used an alpha level set at *p*<0.05. This intervention trial was designed to compare pre- and post-exercise intervention variables. Changes from baseline to the end of the intervention were determined by a paired *t* test and one-way analysis of variance.

## Results

Demographic characteristics of study participants are shown in Table 2. The variables of senior fitness, PGI_2_, and TXA_2_ were measured in all participants before the start and after the end of the 12-week aqua exercise and burdock extract intake program.

**Fig. 1:**
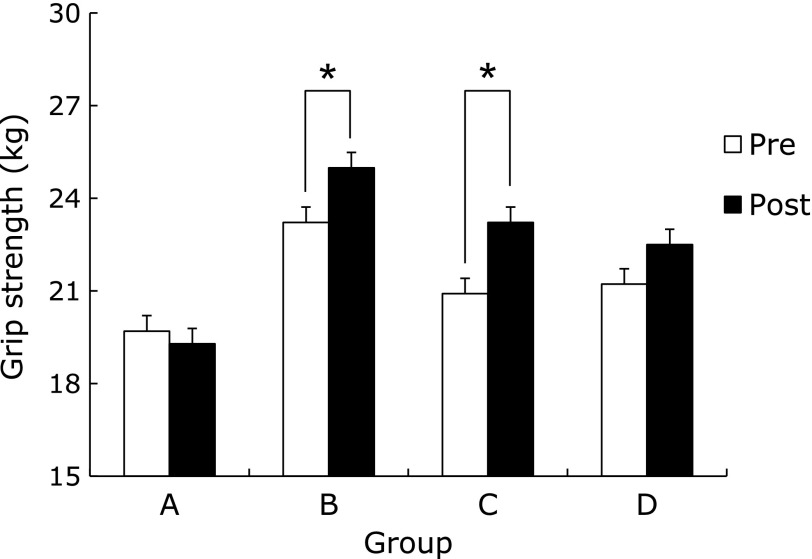
Comparisons of grip strength between pre and post aqua exercise and burdock extract intake. A (CG): Control Group, B (AEG): Aqua Exercise Group, C (AEBG): Aqua Exercise and Burdock extract intake Group, D (BG): Burdock extract intake Group. *p<0.05.

**Fig. 2:**
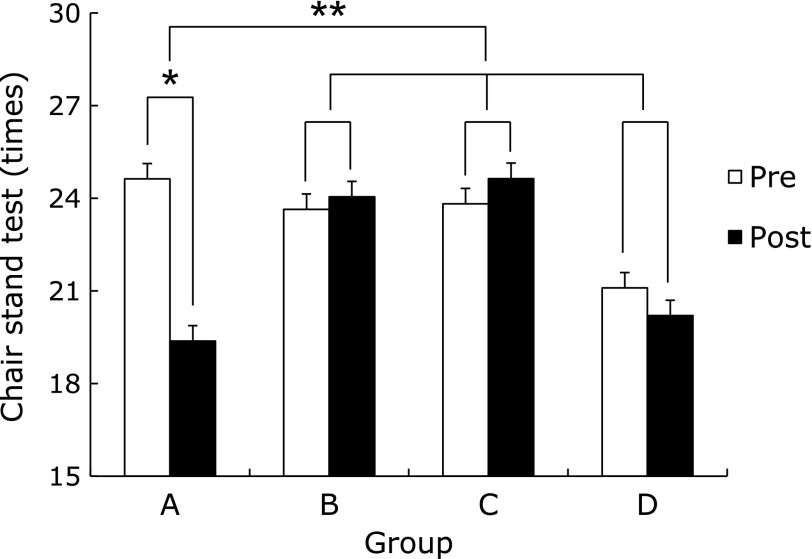
Comparisons of chair stand test between pre and post aqua exercise and burdock extract intake. A (CG): Control Group, B (AEG): Aqua Exercise Group, C (AEBG): Aqua Exercise and Burdock extract intake Group, D (BG): Burdock extract intake Group. *p<0.01, **p<0.001.

Muscular strength of the AEG and AEBG at post-test significantly increased compared to pre-test (*p*<0.05, *p*<0.05) (Fig. 1). The muscular endurance of the CG at post-test significantly increased compared to pre-test (*p*<0.01). There was a significant difference in the rate of muscle endurance change among groups (*p*<0.001), (BG, AEG, and AEBG>CG) (Fig. 2).

**Fig. 3:**
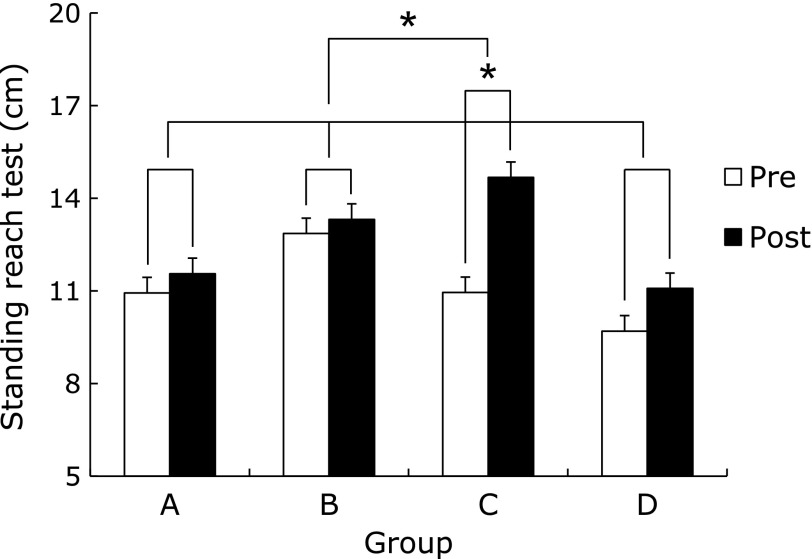
Comparisons of standing reach test between pre and post aqua exercise and burdock extract intake. A (CG): Control Group, B (AEG): Aqua Exercise Group, C (AEBG): Aqua Exercise and Burdock extract intake Group, D (BG): Burdock extract intake Group. *p<0.05.

**Fig. 4:**
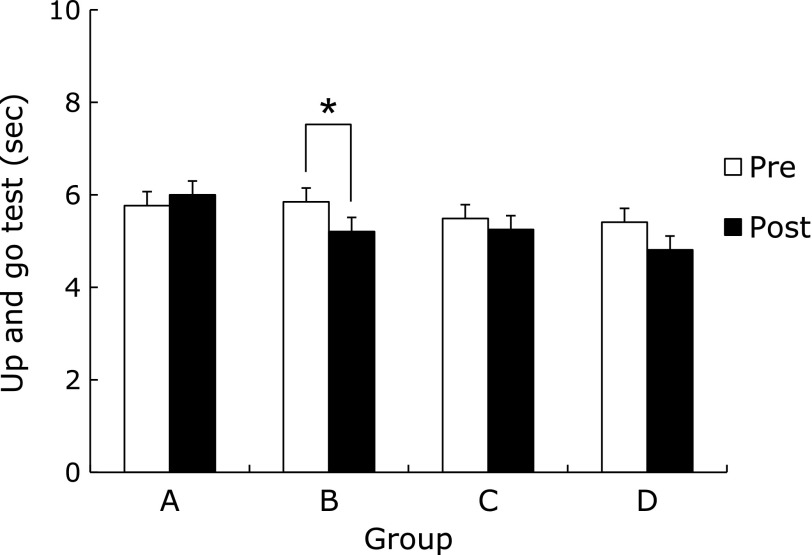
Comparisons of up and go test between pre and post aqua exercise and burdock extract intake. A (CG): Control Group, B (AEG): Aqua Exercise Group, C (AEBG): Aqua Exercise and Burdock extract intake Group, D (BG): Burdock extract intake Group. *p<0.05.

**Fig. 5:**
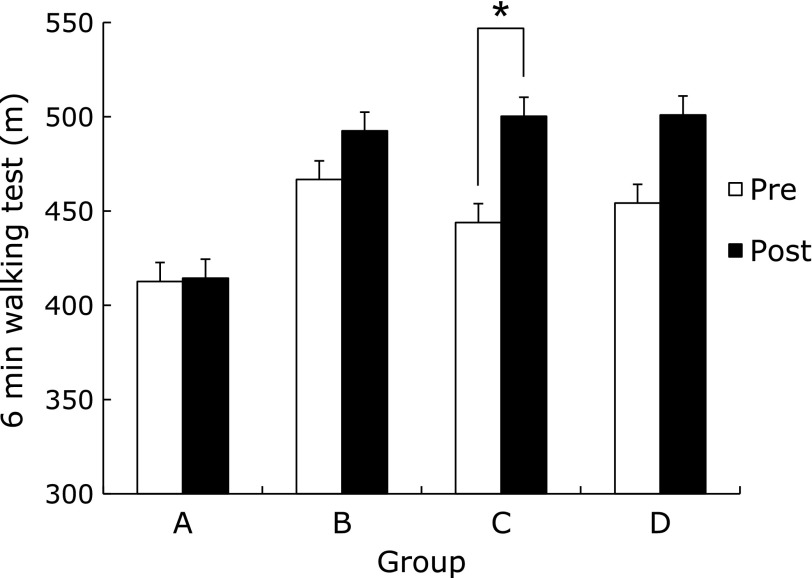
Comparisons of 6 min walking test between pre and post aqua exercise and burdock extract intake. A (CG): Control Group, B (AEG): Aqua Exercise Group, C (AEBG): Aqua Exercise and Burdock extract intake Group, D (BG): Burdock extract intake Group. *p<0.05.

The flexibility of the AEBG at post-test significantly increased compared to pre-test (*p*<0.05). There was a significant difference in the rate of flexibility change among groups (*p*<0.05), (AEBG>CG, AEG, and BG) (Fig. 3). Agility and balance of the AEG at post-test significantly decreased compared to pre-test (*p*<0.05) (Fig. 4). The cardiorespiratory endurance of the AEBG at post-test significantly increased compared to pre-test (*p*<0.05) (Fig. 5).

There were no significant differences in PGI_2_ and TXA_2_ between pre- and post-training programs. But PGI_2_ and TXA_2_ kept the equilibrium. Data are summarized in Table 3 and 4.

## Discussion

Aqua exercise is more effective than non- aqua exercise in terms of weight loss from body fat decomposition because of its burns fat at about twice the rate of non-aqua exercise, despite burning fewer calories per minute.^(28)^ Moreover, it has been reported that aqua exercise is effective at enhancing muscular strength and losing weight and body fat as it applies constant water resistance to joints and muscles.^(29,30)^ In particular, for elderly individuals, aqua exercise is not only effective at reducing the pressure on joints and, thus, pain, because it reduces loads on body parts but also advantageous at reducing risks associated with exercising.^(31,32)^

According to previous studies, a 50–60 min aqua fall-prevention exercise three times a week for 24 weeks improved muscular strength of elderly women aged 65 and older,^(33)^ and 50 min aqua exercise with the intensity of RPE 11–13 three times a week for eight weeks increased dynamic muscular strength in women in late 50s through 60s.^(31,34)^ Tsourlou *et al*.^(35)^ reported that the 24-week 20 min aqua exercise consisting of the endurance exercise with the intensity of 80% maximum heart rate (HRmax) and the upper and lower body resistance exercise with the equipment designed for muscular strength exercise increased static and dynamic muscular strength in healthy elderly women.

The results of the present study were consistent with the findings of previous studies as muscular strength increased significantly in the AEG and AEBG. This result is likely attributable to the improved ability to perform exercise and the increased range of motion in the joints associated with the positive change in body composition.

According to previous studies, 60 min aqua exercise with the intensity of 70% HRmax three times a week for 24 weeks improved muscular endurance in both elderly men and women aged 65–75,^(36,37)^ and 90 min aqua fall-prevention exercise twice a week for 13 weeks increased muscular endurance of the upper and lower limbs in elderly individuals aged 60 and older.^(33)^

The results of the present study show that muscle endurance was significantly lower in the CG than in other groups and tended to increase, although statistically insignificantly, in the AEG and AEBG. This result supports the finding of previous studies that regular exercise increases muscular endurance. The negative result in the muscular endurance of the CG stresses the importance of exercise.

According to previous studies, a 12 week aqua exercise program with the intensity of 50–60% HRmax improved the flexibility of hemiplegic elderly men,^(38,39)^ and 60 min aqua exercise three times a week for 24 weeks increased flexibility in elderly women aged 65–75.^(37)^

The results of the present study show a significant increase of flexibility in the AEBG, and the flexibility was also significantly higher in that group than in the other three groups, supporting the findings of previous studies. This likely suggests the positive effect of aqua exercise and burdock extract intake in improving the range of motion in the joints.

According to previous studies, 60 min aqua exercise three times a week a week for 12 weeks increased balance in elderly women aged 60 and older with osteoarthritis,^(40)^ and 60 min aqua rehabilitation twice a week for 16 weeks improved static balance in elderly individuals with chronic degenerative knee osteoarthritis.^(41)^ Baena-Beato *et al*.^(42)^ also reported that aqua exercise three times a week for eight weeks increased agility in elderly individuals aged 65 and older.

The results of the present study show that agility and balance increased significantly in the AEG and tended to increase, although statistically insignificantly, in the AEBG and BG, in contrast to the decrease in agility and balance in the CG. This is likely a result of the positive change in body composition and overall improvement in physical fitness and functioning due to aqua exercise and burdock extract intake.

According to previous studies, 60 min aqua exercise three times a week for 12 weeks improved cardiorespiratory endurance in women aged 65–75,^(43)^ and 60 min aqua exercise with the intensity of VO_2_max 50–70% three times a week for 12 weeks increased cardiorespiratory endurance in elderly women.^(44)^

The results of the present study show that cardiorespiratory endurance increased significantly in the AEBG, and tended to increase, although statistically insignificantly, in the AEG and the BG, supporting the findings of previous studies. This result likely reflects the positive effect of the sustained aqua exercise and burdock extract intake on improving physical fitness and functioning.

The findings discussed so far indicate that aqua exercise and burdock extract intake have a positive effect on health-related fitness, highlighting the importance of aqua exercise for elderly women and suggesting the potential of burdock as a dietary supplement for enhancing health-related fitness.

Blood vessels voluntarily regulate their expansion and contraction to regulate blood distribution and flow to respond to physiological and chemical stimuli,^(45)^ and the function of blood vessels is reduced drastically after 45 years of age.^(46)^

Healthy endothelial cells secrete PGI_2_, which acts as a vasodilator and a platelet aggregation inhibitor.^(10,47)^ However, in the event of blood vessel damage, endothelial cells separate the blood from collagen, a potential platelet-activating factor, thus preventing platelet aggregation in the healthy endothelial cells.^(46,48)^ The endothelial cells secrete PGI_2_, which inhibits platelet aggregation, and activated PGI_2_ generates TXA_2_ and forms a platelet plug.^(48)^

PGI_2_ is a bioactive substance and performs various functions such as relaxation of vascular smooth muscle and platelet aggregation inhibition. Change in PGI_2_ secretion results in vascular functions in various organs, and diagnostic significance has not been clearly established. Its balance with other factors such as TXA_2_ has particular importance, and the reduction in its secretion increases the risk of diabetes, angina pectoris, cerebral thrombosis, arteriosclerosis, and hypercholesterolemia.^(49)^

TXA_2_ maintains homeostasis in blood vessels through mutually antagonistic action with PGI_2_. TXA_2_ has a strong prothrombic property and is produced by the enzyme reactions that involve activation of phospholipase A_2_, cyclooxygenase, and thromboxane synthase. In the synthetic regulation of TXA_2_, phospholipase A_2_, which generates an arachidonic acid from membranes of phospholipids, plays an important role.^(50,51)^

The results of the present study show no changes in PGI_2_ and TXA_2_ levels in any group; however, the program participation led to balance between PGI_2_ and TXA_2_, which is consistent with the finding of Zoladz *et al.*^(52)^ Considering that both substances are extremely unstable, each with a very short half-life, the positive effects of aqua exercise and burdock extract intake in achieving balance between PGI_2_ and TXA_2_ and maintaining them within the normal range can be viewed as a significant finding.

## Conclusion

Regular and continuous aqua exercise was effective at improving the senior fitness and cardiovascular risk factors of elderly women exposed to decreased muscle mass decreased cardiopulmonary function, and lower cardiovascular disease risk, all of which are due to decreased physical activity. And PGI_2_ and TXA_2_ kept the equilibrium. Additionally, the intake of burdock extracts in vascular health by serving a secondary role in disease prevention and health promotion.

## Figures and Tables

**Table 1:** The main ingredients of Burdock extract

Water (%)	Crude ash (%)	Crude fat (%)	Crude protein (%)	Crude fiber (%)	Calcium (%)	Phosphorus (%)
98.02 ± 0.02	0.10 ± 0.00	1.12 ± 0.00	0.20 ± 0.00	0.03	0.004 ± 0.00	0.009 ± 0.00

(Feed & Foods Nutrition Research Center, Pukyong National University, Busan, Republic of Korea)

**Table 2:** Physical characteristics of subjects in each group

Variables	Age (years)	Weight (kg)	Height (cm)	BMI (kg/m^2^)	% body fat (%)
Groups					
A (*n* = 8)	76.00 ± 5.52	57.68 ± 1.78	152.38 ± 4.31	29.11 ± 12.47	35.41 ± 2.86
B (*n* = 11)	74.09 ± 4.21	56.44 ± 7.29	154.45 ± 4.16	23.92 ± 1.82	39.02 ± 6.23
C (*n* = 11)	74.64 ± 4.59	60.91 ± 7.41	151.90 ± 2.77	26.39 ± 3.26	38.23 ± 4.16
D (*n* = 10)	74.11 ± 4.65	62.17 ± 8.79	154.78 ± 4.94	25.94 ± 2.38	35.50 ± 3.69

Values are Means ± SD. A (CG): Control Group, B (AEG): Aqua Exercise Group, C (AEBG): Aqua Exercise and Burdock extract intake Group, D (BG): Burdock extract intake Group.

**Table 3:** %diff values of PGI_2_ and TXA_2_ in each group

Variable	Group	%diff	*F* value	Duncan
PGI_2_ (pg/ml)	A (*n* = 8)	8.27 ± 14.42	0.367	
	B (*n* = 11)	6.83 ± 25.35		
	C (*n* = 11)	0.25 ± 18.65		
	D (*n* = 10)	0.55 ± 23.98		

TXA_2_ (pg/ml)	A (*n* = 8)	7.92 ± 16.91	0.691	
	B (*n* = 11)	–0.48 ± 12.03		
	C (*n* = 11)	12.04 ± 31.96		
	D (*n* = 10)	7.02 ± 21.06		

Values are Means ± SD. A (CG): Control Group, B (AEG): Aqua Exercise Group, C (AEBG): Aqua Exercise and Burdock extract intake Group, D (BG): Burdock extract intake Group.

**Table 4:** Changes of PGI_2_ and TXA_2_ between pre and post aqua exercise and burdock extract intake

Variable	Group	Pre	Post	*t* value
PGI_2_ (pg/ml)	A (*n* = 8)	19.73 ± 5.54	21.44 ± 7.17	–1.516
	B (*n* = 11)	19.34 ± 8.35	19.88 ± 7.24	–0.33
	C (*n* = 11)	20.51 ± 7.32	20.88 ± 9.49	–0.245
	D (*n* = 10)	20.23 ± 7.39	19.65 ± 6.68	0.308

TXA_2_ (pg/ml)	A (*n* = 8)	21.48 ± 6.44	22.68 ± 5.89	–0.857
	B (*n* = 11)	20.97 ± 6.81	20.99 ± 7.72	–0.015
	C (*n* = 11)	21.55 ± 8.14	24.21 ± 11.49	–1.228
	D (*n* = 10)	19.81 ± 6.33	21.20 ± 6.05	–1.665

Values are Means ± SD. A (CG): Control Group, B (AEG): Aqua Exercise Group, C (AEBG): Aqua Exercise and Burdock extract intake Group, D (BG): Burdock extract intake Group.

## Abbreviations

ETA: eicosatetraenoic
HRmax: heart rate maximum
PGI_2_: prostaglandin I_2_
RPE: rating of perceived exertion
TXA_2_: thromboxane A_2_
